# Calcium Sensing Receptor as a Novel Mediator of Adipose Tissue Dysfunction: Mechanisms and Potential Clinical Implications

**DOI:** 10.3389/fphys.2016.00395

**Published:** 2016-09-08

**Authors:** Roberto Bravo-Sagua, Pamela Mattar, Ximena Díaz, Sergio Lavandero, Mariana Cifuentes

**Affiliations:** ^1^Institute of Nutrition and Food Technology, University of ChileSantiago, Chile; ^2^Faculty of Chemical and Pharmaceutical Sciences and Faculty of Medicine, Advanced Center for Chronic Diseases and Center for Molecular Studies of the Cell, University of ChileSantiago, Chile; ^3^Cardiology Division, Department of Internal Medicine, University of Texas Southwestern Medical CenterDallas, TX, USA

**Keywords:** CaSR, inflammation, adipose tissue dysfunction, obesity, adipocyte, preadipocyte

## Abstract

Obesity is currently a serious worldwide public health problem, reaching pandemic levels. For decades, dietary and behavioral approaches have failed to prevent this disease from expanding, and health authorities are challenged by the elevated prevalence of co-morbid conditions. Understanding how obesity-associated diseases develop from a basic science approach is recognized as an urgent task to face this growing problem. White adipose tissue (WAT) is an active endocrine organ, with a crucial influence on whole-body homeostasis. WAT dysfunction plays a key role linking obesity with its associated diseases such as type 2 diabetes mellitus, cardiovascular disease, and some cancers. Among the regulators of WAT physiology, the calcium-sensing receptor (CaSR) has arisen as a potential mediator of WAT dysfunction. Expression of the receptor has been described in human preadipocytes, adipocytes, and the human adipose cell lines LS14 and SW872. The evidence suggests that CaSR activation in the visceral (i.e., unhealthy) WAT is associated with an increased proliferation of adipose progenitor cells and elevated adipocyte differentiation. In addition, exposure of adipose cells to CaSR activators *in vitro* elevates proinflammatory cytokine expression and secretion. An increased proinflammatory environment in WAT plays a key role in the development of WAT dysfunction that leads to peripheral organ fat deposition and insulin resistance, among other consequences. We propose that CaSR may be one relevant therapeutic target in the struggle to confront the health consequences of the current worldwide obesity pandemic.

## Introduction

Obesity has turned into a pandemic disease, with a worldwide prevalence that has more than doubled in the last three decades, even after multiple attempts to stop its expansion^1^. In 2014, more than 1.900 billion adults were overweight, a number that comprises more than 600 million obese individuals^1^. Besides being associated with disorders such as type 2 diabetes mellitus, hypertension, cardiovascular disease, and cancer (Guh et al., 2009), obesity in itself is a death risk factor (Flegal et al., 2013). There is no doubt that obesity reduces the quality of life and affects the world economic development and productivity (Williams et al., 2005; Slagter et al., 2015).

For decades, many investigations have focused on identifying primary causes, preventive measures, and treatments for halting obesity. Despite these efforts, the long-term impact has been very small (Hafekost et al., 2013), and clinical trials testing different lifestyle-oriented approaches have consistently yielded disappointing results (Langeveld and DeVries, 2015; Ross et al., 2015; Mason et al., 2016). Moreover, the epidemiology reveals that governmental public health interventions focused on diet and physical activity have not been able to decrease the prevalence of obesity or even slow down its increase (Popkin et al., 2012; Cabrera Escobar et al., 2013; Hawkes et al., 2015). Pharmacological approaches have also failed to provide safe and efficacious therapies with long-term relevant results (Yanovski and Yanovski, 2014; Balaji et al., 2016). Considering this scenario, it is clear that there is an urgent need for a deeper understanding of the development of the obesity-associated diseases, and the study of adipose tissue plays a pivotal role in this sense. As written by Elmquist and Scherer (2012), “The solution for the obesity epidemic might lie in better understanding adipocyte biology.” It is now known that white adipose tissue (WAT) dysfunction is key in the pathophysiology of obesity-related diseases, and the study of novel regulators of this process is crucial to uncover new therapeutic targets. In this context, our group showed the presence of the Calcium-sensing receptor (CaSR) in human preadipocytes and adipocytes (Cifuentes et al., 2005), and studies in the last decade suggest that its activation is involved in WAT dysfunction (Villarroel et al., 2014). The present review describes the evidence and perspectives of the role of CaSR in WAT and obesity, as a new player in this complex and multifactorial disorder.

## White adipose tissue: a key homeostatic organ

**Figure 1:**
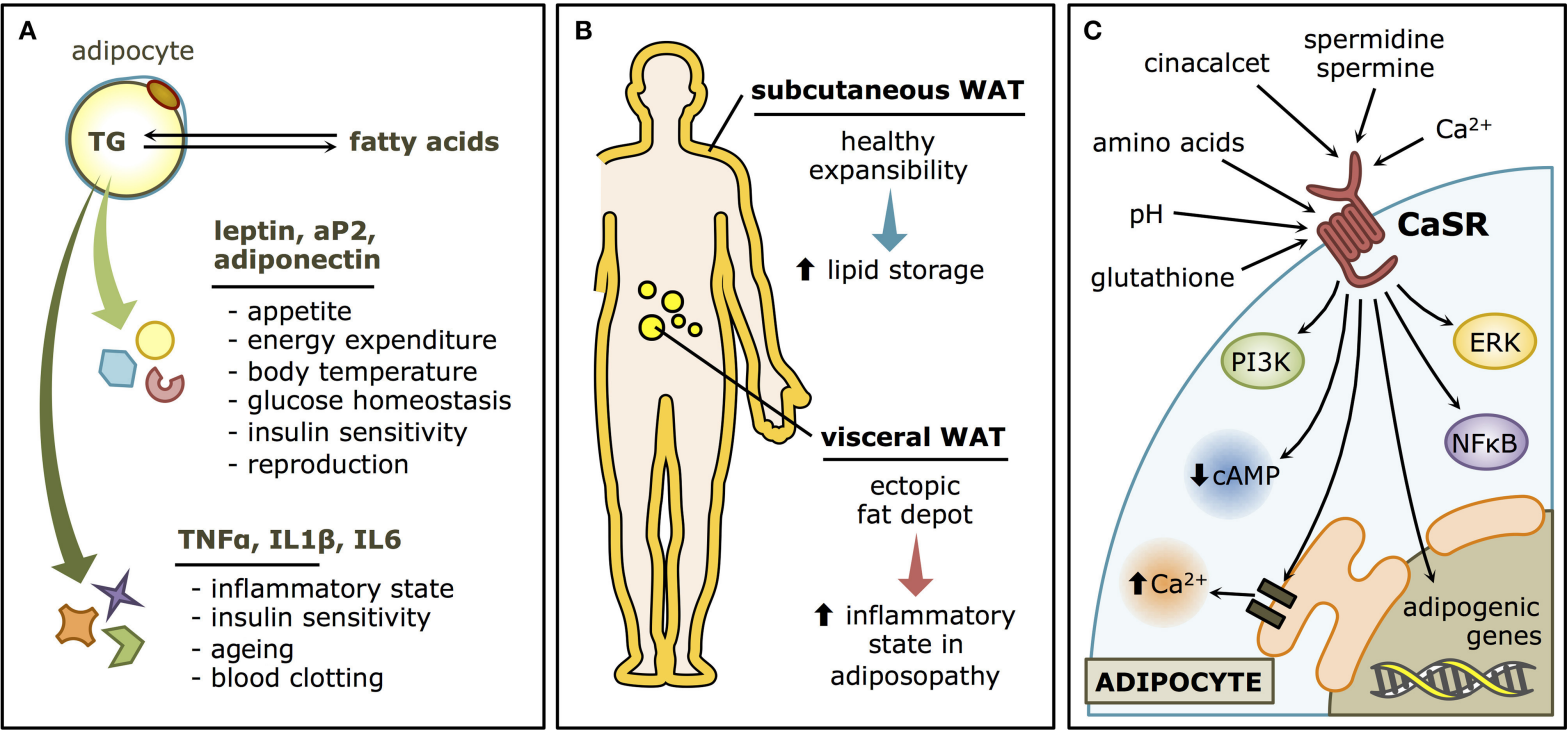
**White adipose tissue (WAT) and the Calcium-sensing receptor (CaSR). (A)** Functions of the WAT. Adipocytes store lipids in the form of triglycerides and release them as fatty acids. The WAT also has an endocrine role, through the secretion of adipokines such as leptin, adiponectin and aP2 that regulate whole-body metabolism, and cytokines such as TNFα, IL1β, and IL6, with local and distal modulatory functions, that may determine the “low grade inflammatory status” that characterize most obese patients. **(B)** Types of WAT. Subcutaneous WAT is considered rather innocuous (or even beneficial) and has an important expansibility potential in healthy individuals. Visceral WAT, on the other hand, is a major player in adiposopathy, as it contributes to the inflammatory state that characterizes WAT dysfunction. **(C)** CaSR signaling pathways in WAT. CaSR has both physiological activators, such as Ca^2+^ and polyamines, and pharmacological modulators, such as cinacalcet, as well as a variety of allosteric regulators associated with metabolism, such as pH, amino acids, and glutathione. In the adipocyte, the canonical CaSR-associated pathways are thought to be activated, like ERK, PI3K, NFκB, and Ca^2+^ elevations and cAMP decreases through Gα proteins. The adipogenic program is also known to be stimulated by CaSR stimulation.

WAT is currently regarded as a dynamic organ with an extraordinary capacity to expand or decrease, according to the energy status of the organism (Pellegrinelli et al., 2016). There is considerable interest in studying WAT due to its relevance as an endocrine organ and as a whole-body metabolic regulator, particularly in light of the current obesity epidemic. The main functional component of WAT is the adipocyte, which specializes in storing energy as triglycerides and releasing it as fatty acids. The tissue is also composed of the so-called stroma-vascular fraction, which contains adipocyte progenitor cells (preadipocytes), and fibroblasts, as well as endothelial, smooth muscle, and immune cells. Besides its storage function, WAT also regulates whole-body energy homeostasis through the production of regulatory paracrine/endocrine molecules, termed adipokines (Rosen and Spiegelman, 2014). These secretory products control a wide variety of biological functions (Figure 1A), such as appetite, energy expenditure, body temperature, glucose homeostasis, insulin sensitivity, inflammation, blood clotting, reproduction, and ageing (Berry et al., 2013; Hyvönen and Spalding, 2014).

It is well-accepted that body fat distribution largely determines whether metabolic and cardiovascular comorbidities develop in an obese individual, favoring their occurrence when fat accumulation occurs at the abdominal level (“central obesity”; Jensen, 2008). The subcutaneous and the visceral compartments (Figure 1B) have very different clinical implications. Proliferation of the subcutaneous adipose tissue is considered positive, as it leads to increased “healthy” storage capacity (Gustafson et al., 2015). Inflammatory cues associated with obesity lead to impaired expansibility of the subcutaneous depots (Tchernof and Després, 2013). As a consequence, enlargement of visceral preadipocytes drives more inflammation and the increase of ectopic fat depots (Smith, 2015), associated with pathologic effects (Gustafson et al., 2009; Ye and Gimble, 2011). Ectopic fat accumulation leads to lipotoxicity where fatty acids are accumulated in peripheral tissues, impairing cellular signaling, and functions (Lee et al., 2013), constituting what has been termed “adiposopathy”(Bays, 2014).

Adiposopathy takes place when obesity-triggered changes, such as adipocyte hypertrophy and lipid overload, prevent WAT from properly performing its storage and endocrine functions (Gustafson et al., 2009). Dysfunctional adipocytes develop an overall inflammatory state, secreting cytokines that result in the infiltration of macrophages, which in turn produce higher amounts of proinflammatory cytokines. Such environment further compromises normal adipocyte function, particularly triglyceride deposition (Guilherme et al., 2008). This results in increased circulating free fatty acids and ectopic fat accumulation, ultimately leading to insulin resistance, and functional impairment in other organs, especially skeletal muscle.

## CaSR: a role in adipose tissue dysfunction

**Figure 2:**
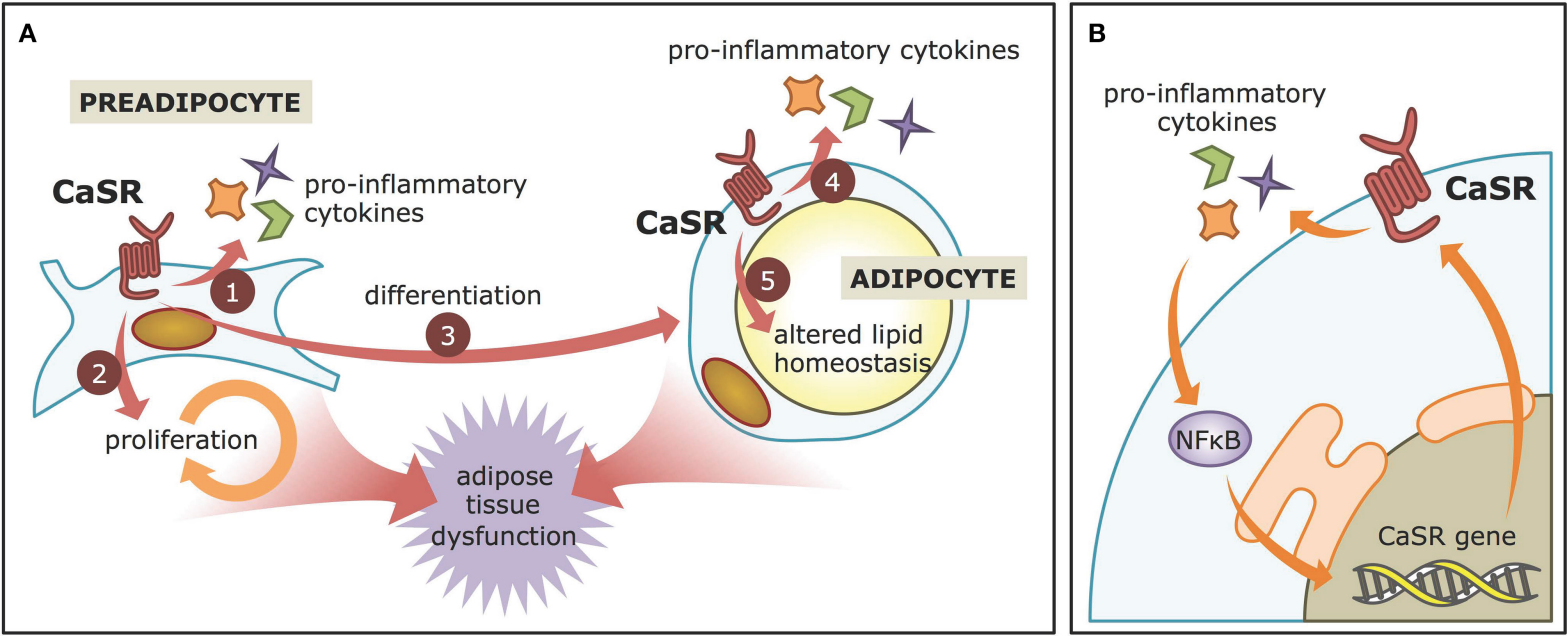
**CaSR contributions to adiposopathy. (A)** CaSR activation in visceral WAT preadipocytes leads to increased production of pro-inflammatory cytokines, proliferation, and differentiation. In adipose cell models, it also enhances pro-inflammatory cytokine production and may decrease lipid accumulation, thereby contributing to adipose tissue dysfunction. **(B)** The NFκB-CaSR positive feed-back. CaSR activation leads to increased pro-inflammatory cytokines secretion, which are known to activate the NFκB pathway. NFκB, in turn, stimulates CaSR gene expression via specialized sequences in the CaSR promoter.

CaSR is an extracellular Ca^2+^ sensor, originally described in the parathyroid gland as a regulator of parathyroid hormone secretion and circulating Ca^2+^ levels (Brown et al., 1993; Garrett et al., 1995). CaSR is a G-protein coupled receptor with 7 transmembrane helices and a very complex signaling network (Figure 1C) comprising orthosteric and allosteric modulators (Cavanaugh et al., 2012). Among the many non-Ca^2+^-homeostatic roles that have been described for the CaSR, its involvement in WAT physiology emerged since its presence in human WAT was reported in 2005 (Cifuentes et al., 2005). CaSR activation in WAT is associated, by different mechanisms, with alterations consistent with a dysfunctional phenotype (Figure 2A). Activation of CaSR in human adipose cells, as well as WAT explants, elevates the expression of the proinflammatory cytokines interleukin 6 (IL6), chemokine C-C motif ligand 2 (CCL2), interleukin 1β (IL1β), and tumor necrosis factor alpha (TNFα; Cifuentes et al., 2012), which have been linked with adipose dysfunction and the cardiovascular and metabolic consequences of obesity. In addition, CaSR activation stimulates the proliferation, and proinflammatory cytokine expression in preadipocytes (Rocha et al., 2015). Moreover, CaSR activation elevates adipogenesis in visceral preadipose cells (Villarroel et al., 2013).

### Pro-inflammatory cytokine expression

Several reports have described how CaSR activation is linked to inflammation in adipose cells, which is the cardinal feature of WAT dysfunction. The human adipose cell line LS14, as well as primary preadipocytes, and adipocytes, not only express CaSR, but also upregulate its protein levels in response to pro-inflammatory cytokines, such as IL1β, IL6, and TNFα (Cifuentes et al., 2010). Furthermore, adipocytes treated with conditioned medium of WAT explants obtained from obese individuals also increase their CaSR protein levels. These elevations at least in part depend on NFκB signaling pathway, as its inhibitor, SN50, reduces the effect. These findings are consistent with previous work showing the presence of NFκB response elements in the CaSR promoter (Canaff and Hendy, 2005). The CaSR, in turn, increases the secretion of proinflammatory cytokines in adipose cells (Cifuentes et al., 2012), possibly resulting in a positive feedback loop. CaSR activation with the calcimimetic cinacalcet increases the expression of IL1β, IL6, TNFα, and CCL2 in LS14 preadipocytes and adipocytes as well as human visceral WAT explants. Again, these changes were dependent on NFκB signaling in LS14 cells (Cifuentes et al., 2012), thus highlighting the intimate, tripartite relationship between CaSR-NFκB-cytokines during inflammation in adipose cells (Figure 2B).

### Higher visceral adipogenesis

Studies in both SW872 and LS14 human adipose cell lines have revealed an adipogenic effect of CaSR (He et al., 2012; Villarroel et al., 2013). Exposure to CaSR activators increases the mRNA levels of peroxisome proliferator-activated receptor γ (PPARγ), a master regulator of the adipogenic genetic program, thereby upregulating its downstream genes, such as adipose fatty acid-binding protein (aP2), lipoprotein lipase (LPL), CCAAT element binding protein α (C/EBPα), glycerol-3-phosphate dehydrogenase (GPD), and adiponectin. In response to body fat overload, healthy subcutaneous adipogenesis is considered a positive measure to increase storage capacity. However, as aforementioned, not all fat depots are equal, and increased visceral WAT is associated with inflammation and obesity-induced cardiovascular and metabolic impairment. In the case of LS14 cells, they are considered a model of visceral adipocytes (Hugo et al., 2006; LaPensee et al., 2008). Given their proinflammatory profile elicited by CaSR activation, this kind of adipogenesis rather contributes to adiposopathy, instead of its prevention or relief.

### Preadipocyte proliferation

In both human LS14 and murine 3T3L1 preadipocytes, different CaSR agonists increase proliferation through the ERK signaling pathway (Hou et al., 2013; Rocha et al., 2015). This mitogenic effect of CaSR activation has also been shown in other cell types, such as rat bone marrow mesenchymal stem cells (Xu et al., 2012), and rat osteoblasts (Chattopadhyay et al., 2004). Moreover, the involvement of the CaSR-ERK signaling axis is consistent with findings in breast cancer cells (El Hiani et al., 2009). As is the case for interpreting the adipogenesis observations, being considered a model of visceral WAT, LS14 proliferation may be linked to the pathologic effects of obesity, instead of a healthy subcutaneous WAT remodeling.

### Altered lipid handling

Numerous epidemiological, clinical, and cell-based studies have suggested that a diet deficient in calcium is associated with greater fat accumulation (Villarroel et al., 2014). The proposed mechanism involves paradoxically greater cytosolic calcium levels in adipocytes with low calcium diets that trigger changes in triglyceride metabolism (Zemel and Miller, 2004). Interestingly, one of the main downstream signaling events triggered by CaSR activation is elevated cytosolic Ca^2+^, and rats fed low calcium diets showed an elevated expression of CaSR in WAT (He Y. H. et al., 2011). In addition, the low calcium diet was associated with a decrease in triglyceride breakdown (lipolysis) in rat WAT, which was dependent on CaSR (He Y. H. et al., 2011). The low calcium diet also decreased protein levels of the lipolytic enzymes hormone-sensitive lipase and adipose triglyceride lipase. Using the SW872 human adipose cell line, this CaSR-mediated antilipolytic effect was shown to rely on intracellular Ca^2+^ augmentation, as well as a decrease in cyclic AMP (cAMP) and cAMP-dependent protein kinase A (PKA) signaling (He Y. et al., 2011). Similar results were obtained in primary human adipocytes, where CaSR activation decreased lipolysis via Giα protein and phosphoinositide 3 kinase (PI3K) signaling (Cifuentes and Rojas, 2008). Taken together, the described findings suggest that even though CaSR stimulates WAT proliferation and differentiation, it induces alterations in lipid handling that might contribute to the deleterious effects of obesity. In this context, it may be of great interest to perform controlled trials to evaluate possible changes in visceral adiposity and serum lipid profiles in patients undergoing treatment with cinacalcet.

### Integrative model

Among the many regulators of the CaSR (reviewed in Cavanaugh et al., 2012), the polyamines spermine and spermidine are two orthosteric CaSR agonists with promising roles regarding WAT metabolism. Obese Zucker rats have four times more spermine and spermidine in the adipose tissue in comparison with lean animals, which correlates with increased activity of various triacylglycerol synthetic enzymes (Jamdar et al., 1996). Aside from polyamines, other metabolic indicators potentiate CaSR signaling, such as pH elevations (Doroszewicz et al., 2005), glutathione (Wang et al., 2006), and amino acids (Lee et al., 2007), thus supporting the possibility that outside of the parathyroid gland, CaSR acts more like a metabolic status sensor instead of a Ca^2+^ rheostat. Due to CaSR's unique ability to sense, and thus potentially integrate a variety of signals through distinct allosteric sites, it may be considered a sensor of the local metabolic environment (Breitwieser et al., 2004), which is of great interest in complex tissues, or pathogenic contexts such as dysfunctional WAT and obesity. Additionally, CaSR folding and traffic have been indicated as key regulators of its activity (Grant et al., 2011). This is interesting given that in obesity, adipocytes are subjected to endoplasmic reticulum (ER) stress (Kawasaki et al., 2012), which specifically compromises protein folding and traffic. Moreover, CaSR synthesis in the ER includes a folding checkpoint and retention step (Cavanaugh et al., 2010), which can be assisted with agonists such as Ca^2+^ and glutathione (Breitwieser, 2014). Considering that adiposopathy leads to oxidative stress and altered glutathione metabolism in adipocytes (Kobayashi et al., 2009), we propose CaSR as a novel potential integrator of environmental cues, which ultimately contribute to adipose tissue dysfunction and pathology.

## Future studies

Ongoing studies of CaSR in WAT pathophysiology are contributing to elucidate an important role of the receptor, mainly in the visceral depot or in models of visceral adipose cells. Given the fundamental differences between WAT depots, it is of great relevance that future studies begin to address the role of the receptor in the subcutaneous fraction. Moreover, studying the expression and function of the CaSR in obese and lean individuals, as well as healthy versus unhealthy obese, and exploring whether there exists a sexual dimorphism in WAT CaSR expression and function, are relevant pending issues.

For a better insight on the role of CaSR in the pathogenesis of obesity, studies should also test the effect of CaSR modulation in different obesity models, such as diet-induced and genetically modified mice. Given the function of CaSR in multiple tissues, as well as its preponderance in regulating circulating calcium, it should be considered to modulate its function locally at the WAT. Additionally, its role during the onset and progression of fat accumulation should be tested. Moreover, the WAT of patients with activating or downregulating mutations in the CaSR should be analyzed, in order to detect disturbances in their metabolic or inflammatory state.

In recent years, not only WAT, but also brown adipose tissue (BAT) has received increasing attention. BAT is a type of adipose tissue characterized for being thermogenic and rather metabolically active instead of storage-oriented (Pellegrinelli et al., 2016). Different reports have shown its presence and activity in healthy adult humans, where up to recent years it was thought to be irrelevant (Cypess et al., 2009; Saito et al., 2009; van Marken Lichtenbelt et al., 2009; Virtanen et al., 2009). BAT is a promising target in the treatment of metabolic diseases, as it inversely correlates with body weight and fasting circulating glucose (Lee et al., 2010). Cold exposure has been shown to stimulate BAT presence in humans (Hanssen et al., 2016) probably through transdifferentiation of WAT (Frontini et al., 2013). White-to-brown transdifferentiation, commonly termed “browning” or “beigeing,” has been shown to occur only in subcutaneous (Fisher et al., 2012) but not in visceral WAT, further underscoring the differences between both types of adipose tissue and the need to explore the role of CaSR in the subcutaneous depot. Studies are required to assess the role of CaSR in BAT metabolic and regulatory functions, and also to test whether CaSR pro-inflammatory effect negatively affects WAT browning.

## Conclusion

Obesity is a complex and multifactorial disease, and even though it is known that energy imbalance is at the core of the problem, it has been extremely difficult to decrease or control the worldwide obesity epidemic. Current efforts aim to reduce obesity-related diseases by better understanding WAT physiology and maintaining its adequate function. The CaSR in WAT is emerging as an important molecule whose activation has been shown to increase the inflammatory state. Moreover, CaSR activation also promotes the proliferation of WAT under inflammatory conditions, which is known to yield less functional fat depots that rather contribute to pathogenesis than to lipid storage. In this sense, inhibition of CaSR activity in the WAT of obese patients may represent a novel therapeutic approach in this obesity epidemic era. The effects here described are in accordance with recent findings indicating that CaSR activation participates in many inflammatory processes, such as recognition of necrotic cells (Rossol et al., 2012) and pathologies like asthma (Yarova et al., 2015), myocardial infarction (Liu et al., 2015), and sepsis (Wu et al., 2015). On the other hand, CaSR-induced inflammation in the gastrointestinal tract has been proposed to be a conditioning agent for the appropriate function of the intestinal epithelial barrier (Owen et al., 2016). Thus, further research should evaluate the potential impact of CaSR activation as a pharmacological target in the pathogenesis of WAT dysfunction.

## Author contributions

MC and RB designed and outlined the structure and contents of the review. RB, PM, XD, SL, and MC contributed to the literature review, discussion, and writing of the manuscript. All authors contributed equally to the draft revisions and final approval of the version to be published.

### Conflict of interest statement

The authors declare that the research was conducted in the absence of any commercial or financial relationships that could be construed as a potential conflict of interest.

## Footnotes

^1^http://www.who.int/mediacentre/factsheets/fs311/en/.
